# Substituent Effects on the Solubility and Electronic Properties of the Cyanine Dye Cy5: Density Functional and Time-Dependent Density Functional Theory Calculations

**DOI:** 10.3390/molecules26030524

**Published:** 2021-01-20

**Authors:** Austin Biaggne, William B. Knowlton, Bernard Yurke, Jeunghoon Lee, Lan Li

**Affiliations:** 1 Micron School of Materials Science and Engineering, Boise State University, Boise, ID 83725, USA; austinbiaggne@u.boisestate.edu (A.B.); bknowlton@boisestate.edu (W.B.K.); bernardyurke@boisestate.edu (B.Y.); jeunghoonlee@boisestate.edu (J.L.); 2 Department of Electrical and Computer Engineering, Boise State University, Boise, ID 83725, USA; 3 Department of Chemistry and Biochemistry, Boise State University, Boise, ID 83725, USA; 4 Center for Advanced Energy Studies, Idaho Falls, ID 83401, USA

**Keywords:** density functional theory, time dependent density functional theory, cyanine dye, Cy5, exciton, solubility, dipole moment

## Abstract

The aggregation ability and exciton dynamics of dyes are largely affected by properties of the dye monomers. To facilitate aggregation and improve excitonic function, dyes can be engineered with substituents to exhibit optimal key properties, such as hydrophobicity, static dipole moment differences, and transition dipole moments. To determine how electron donating (D) and electron withdrawing (W) substituents impact the solvation, static dipole moments, and transition dipole moments of the pentamethine indocyanine dye Cy5, density functional theory (DFT) and time-dependent (TD-) DFT calculations were performed. The inclusion of substituents had large effects on the solvation energy of Cy5, with pairs of withdrawing substituents (W-W pairs) exhibiting the most negative solvation energies, suggesting dyes with W-W pairs are more soluble than others. With respect to pristine Cy5, the transition dipole moment was relatively unaffected upon substitution while numerous W-W pairs and pairs of donating and withdrawing substituents (D-W pairs) enhanced the static dipole difference. The increase in static dipole difference was correlated with an increase in the magnitude of the sum of the Hammett constants of the substituents on the dye. The results of this study provide insight into how specific substituents affect Cy5 monomers and which pairs can be used to engineer dyes with desired properties.

## 1. Introduction

Dyes in natural [1,2,3] and synthetic [4,5,6,7,8] systems have been shown to exhibit molecular aggregation behavior of which exciton delocalization is a signature. Exciton delocalization can be described as the collective sharing of an electronic excitation over dyes within an aggregate due to the transition dipole-dipole coupling between the dyes. Upon aggregation, the dyes can assume various stacking geometries that can be best described in the context of the simplest dye aggregate—the dimer. Three idealized dimer aggregate stacking geometry cases that are commonly presented, as related to the transition dipole moment of one dye relative to the other dye, are: head to tail (J-aggregates [9,10,11,12]), stacked or face-to-face (H-aggregates [10,11,13]), and oblique, in which the transition dipole moments (polarizations) of dyes are at 90 degrees to one another [10,11,14,15]. The geometric orientation of the transition dipole moments and subsequent transition dipole-dipole coupling manifests as changes in excitonic behavior. Potential applications of excitonic properties of dye aggregates include organic photovoltaics [16], non-linear optics [17], and quantum computing [18,19]. The functionality of excitonic devices utilizing dye aggregate properties is therefore determined by the dynamics of the excitons in the material, such as exciton exchange between the dye aggregates and exciton-exciton interactions.

The dynamics of excitons that exist in an aggregate of molecules are characterized by the Frenkel Hamiltonian [20], which describes the mechanisms and associated energy terms of exciton hopping and exciton-exciton interactions. The exciton exchange energy and two-body exciton interaction energy determine the Hamiltonian. Assuming the dipole approximation, the exciton exchange energy, associated with the transfer of an exciton between two adjacent dyes, is enhanced with increasing transition dipole moments (μ) of the dye monomers. Similarly, the two-body exciton interaction energy, associated with changes in the static dipole-dipole coupling of the dyes with differing ground and excited state charge distributions, increases with increasing static dipole difference (Δ**d**) between the dye monomer’s ground and excited states [20]. Both energy terms also increase as the dye-dye separations decrease. Because of the dependence of the Frenkel Hamiltonian energies on dye monomer properties, enhancement of the aggregate’s excitonic properties can be facilitated by the increase of μ, Δ**d**, and aggregation ability of the dye monomers.

Because of the strong excitonic coupling leading to exciton delocalization found in cyanine dye aggregates, the structural, photophysical, and excitonic properties of polymethine indocyanine dyes, such as Cy3 and Cy5, incorporated into DNA structures have been studied experimentally [7,8,21,22,23,24]. Cy3 and Cy5 both contain polymethine bridges connecting two indolenine groups with a delocalized charge. Cy3 and Cy5 have similar structures, their only difference is the number of carbons in the polymethine bridge (three for Cy3 and five for Cy5). In practice, DNA is used as a scaffold to better control the number and orientation of the dyes and bring them within close enough proximity to induce exciton delocalization. Markova et al. demonstrated that Cy5 dyes internalized in DNA duplexes exhibited J-like absorption properties [21]. Similarly, Cannon et al. measured the absorption of Cy5 dyes incorporated into DNA duplexes and found that at low salt concentrations, Cy5 dyes aggregated in J-dimers [7]. By increasing the salt concentration, the DNA coupled to form tetramer aggregates in which the dyes oriented in H-like aggregates and exhibited strong exciton delocalization. Furthermore, it was found that dyes brought within 2 base-pairs of each other exhibit Davydov splitting [7]. In a later study, Cannon et al. found that Cy5 dyes covalently bound in DNA Holliday Junctions exhibited strong Davydov splitting and exciton delocalization [8]. Cunningham et al. also found that Cy3 dyes brought within 2 base-pairs of each other exhibit Davydov splitting and exciton delocalization and that local DNA sequences in proximity to the dyes affected the resulting dye orientations [22]. Nicoli et al. demonstrated that Cy3 dyes covalently attached to DNA backbones exhibited H-like aggregate properties which could be varied by changing the dye-dye separations and DNA rigidity [23].

Studies have shown that the addition of substituents can alter the permanent molecular dipole moments and aggregation properties of molecules [25,26,27,28]. Misawa et al. measured large changes in the static dipole moments of pseudoisocyanine bromide (PIC-Br) upon J-aggregation, revealing that cyanine molecules had the ability to achieve large static dipole moments [25]. Utilizing Stark spectroscopy, Marder et al. measured large static dipole moments (~40 Debye) of a normally symmetric β-carotene molecule [26]. The large static dipole values were engineered by inducing electronic asymmetry into the β-carotene, which augmented the electron withdrawing ability of one side of the otherwise symmetric molecule. This could also happen to cyanine molecules. Garoff et al. found that alterations of the heterocyclic groups of cationic cyanine dyes yields varying aggregation on DNA [27]. Quinoline substituents outperformed benzothiazole, benzoxazole, and dimethylindole in terms of aggregation ability. By incorporating various substituent types into a cyanine dye, Stadler et al. demonstrated significant substituent effects on dye-aggregation [28]. Methoxy and fluoro groups attached to the ends of the cyanine dyes improved dimerization in an aqueous solution and in DNA.

In previous computational studies, researchers have used density functional theory (DFT) and time-dependent density functional theory (TD-DFT) methods to calculate the structural and electronic properties of organic molecules [29,30,31]. Fothergill et al. successfully used TD-DFT to calculate the vibrationally-resolved Cy5 monomer absorption spectrum that yielded a max absorption energy within 0.007 eV of experimental values [29]. Cao et al. used DFT and TD-DFT to perform a systematic analysis of the effects of an amino group on the electronic properties of Cy3, Cy5, Cy7, and Cy9 dyes when the amino group was placed at different locations along the polymenthinic chain [30]. The amino group substituent altered the geometry and spectral properties of the dyes, yielding a wide range of peak absorption energies [30]. The effects of single, double, and quadruple substitutions of substituents on the excitation properties of anthracene molecules were also explored using DFT and TD-DFT by Abou-Hatab et al. [31]. The addition of substituents to the anthracene molecule overall red-shifted excitation energies and raised oscillator strengths [31]. The excitation energies of modified anthracene were found to be correlated with the Hammett constants of the substituents [31]. Quantum chemical calculations of ground and excited state dipole moments of coumarin [32] and quinoline [33] molecules have also been shown to agree with experimentally derived values.

The purpose of this study is to quantify how substituents affect the solubility and electronic properties of Cy5 dye monomers using DFT-based methods. Dye aggregate simulations using molecular dynamics are ongoing and beyond the scope of this manuscript. In both experimental and computational studies, the impacts of substituents on dyes and their excited state properties are crucial but have not been fully addressed. Specifically, computational studies on the relationships of hydrophobicity, μ and Δ**d** of substituted Cy5 dyes have not been performed. This paper demonstrates that the hydrophobicity and Δ**d** of a Cy5 dye can be altered without degrading μ. The modification of Cy5 hydrophobicity is desirable to promote denser dye packing and the increase of Δ**d** would promote larger exciton-exciton interactions in the Cy5 aggregates. However, to preserve exciton exchange energy, μ should not be decreased upon substitution. To understand the effects of various substituents on the electronic properties of Cy5, substituents with varying electron donating and withdrawing strengths were added to the dye. We performed DFT and TD-DFT calculations for each substituted Cy5 dye to calculate the Gibbs free energy of solvation (ΔG_solv_), Δ**d**, and μ in comparison with pristine (unsubstituted) Cy5. Substituted Cy5 dyes exhibiting large Δ**d** were selected for further calculations, in which the number of substituents was doubled to determine the effects of multiple substituents on the solvation and dipole properties. Please note that our study focuses on the computational screening of potential Cy5 substituents that could increase the Δ**d** value of pristine Cy5 but not decrease its μ value.

## 2. Computational Methods

Excited state properties are dependent on the exchange-correlation functionals chosen [34,35,36,37,38,39]. Jacquemin studied the viability of various exchange-correlation functionals for the ground and excited state calculations of 31 different molecules as well as increasingly long push-pull chains [35]. It was determined that the M06-2X and CAM-B3LYP functionals yielded excess dipoles (synonymous with static dipole differences) that strongly correlated to approximate second order coupled clusters double (CC2) methods [35]. Kawauchi et al. found that the prediction of absorption spectra for a set of organic dyes were more accurately predicted using the M06-2X functional than others [36]. Similarly, Laine et al. found that the M06-2X functional could be used to accurately predict experimental bathochromic shifts of BODIPY dyes [37]. Garcia et al. performed a series of TD-DFT calculations on a family of push-pull compounds and found that, out of the functionals tested, CAM-B3LYP was one of the functionals that yielded the best agreement with experimental vertical absorption energies [38]. Kerkines et al. also found that CAM-B3LYP gave good agreement with experiment for excited state properties for the push-pull organic dye DMA-DPH [39].

Due to the common and successful applications of M06-2X and CAM-B3LYP, both functionals were used for the calculations in the present work for comparison. All dyes were optimized in the ground state using the M06-2X functional [35,36,37,40] to a residual force of 4.5 × 10^−4^ Hartree/Bohr with a validation to be at minima through ground state vibrational analysis. Single point excited state calculations were performed on the optimized ground state structures to obtain vertical transitions to the lowest excited singlet states of the dyes using the M06-2X and CAM-B3LYP functionals. Both are hybrid functionals. However, M06-2X is defined as a global hybrid [40] while CAM-B3LYP is defined as a range-separated hybrid functional [41]. Calculations were performed using the 6 − 31 + g(d,p) basis set due to its common usage in similar studies [37,42,43] and to compromise between accuracy and computational resources. The Gaussian16 ab initio quantum chemistry package was employed [44] and initial molecule structures were built using the GaussView GUI [45].

To determine the effect of substituents on the solubility of Cy5, vacuum and implicit solvation calculations were conducted to determine the ΔG_solv_ of each substituted Cy5 dye. In general, ΔG_solv_ is the amount of energy required to dissolve a dye in solvent. Similar to prior DFT studies [46,47], ΔG_solv_ is correlated to solubility, where the more negative ΔG_solv_ is, the more soluble the molecule [46,47]. To calculate ΔG_solv_, the dyes were fully relaxed in vacuum and solvent in the ground state. The universal Solvation Model based on Density (SMD) [48] variation of the integral equation formalism polarizable continuum model (IEFPCM) [49,50] was used to model the dyes in solvents for solvation energy calculations. Four solvents were used, including water, pyridine, quinoline and isoquinoline. The last three solvents were used to mimic the effect of surrounding DNA in comparison with water solvation due to their similar structures to DNA nucleobases. Pyridine, quinoline, and isoquinoline were used to roughly estimate how various substituents on Cy5 dyes affect the dye’s propensity to intercalate into DNA structures. Despite this being a relatively simple model, it could provide insight into dye-DNA interactions for further studies.

The ΔG_solv_ for pristine and substituted dyes was determined as [29,47]:

ΔG_solv_ = E_solvated_ − E_vacuum_ (1)

where E_solvated_ is the total energy of the dye in implicit solvent and E_vacuum_ is the total energy of the dye in vacuum. A negative ΔG_solv_ signifies that the solvation is exothermic and an amount of energy is released. Calculations were also performed using the Gibbs free energies of the dyes in solvent and vacuum instead of the total energies, similar to Abdur Rauf et al. [46]. However, it was found that the energy corrections were minimal and trends between data sets remained the same, as shown in Figure S1.

Calculations of the ground and excited state electronic properties (i.e., dipole properties) were performed using the IEFPCM solvation model without SMD variation [51,52,53] in water solvent. The ground state optimized structures were used for linear-response, single-point, excited state calculations [54]. To quantify the effects of substituents on μ and Δ**d**, the single point excited state calculations were used to determine excited state dipole moments and μ. Using the static ground state and excited state dipole moments, Δ**d** was calculated as [35]:

Δ**d** = [(**d**_x_^ES^ − **d**_x_^GS^)^2^ + (**d**_y_^ES^ − **d**_y_^GS^)^2^ + (**d**_z_^ES^ − **d**_z_^GS^)^2^]^1/2^ (2)

where **d***_i_^j^* is the Cartesian dipole moment vector component, *i* refers to the Cartesian x, y, or z direction, and *j* is either GS or ES (ground state or excited state).

**Figure 1:**
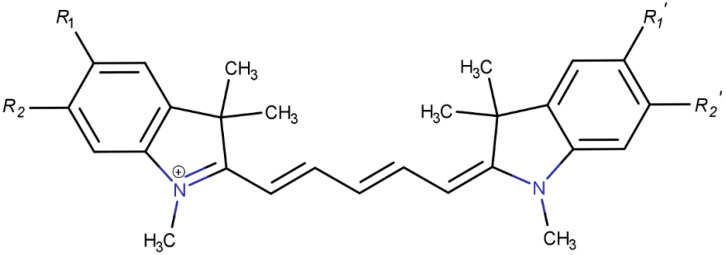
Molecular structure of a Cy5 (1,1′-dimethyl-3,3,3′,3′-tetramethylindocarbocyanine) dye. Hydrogens at ‘R’ locations were replaced with substituents.

Hydrogens at the ends of a Cy5 dye, labeled as R in Figure 1, were replaced with the substituents in Table 1. The substitution of the hydrogens at the R sites produces conjugated systems with increased polarity and asymmetry. As shown in Table 1, each substituent is identified as either a donating or withdrawing group based on its empirically derived Hammett Constant, σ_p_ [55,56]. The magnitude of σ_p_ quantifies the electron donating or withdrawing ability of a substituent. Negative values correspond to electron donating groups and positive values correspond to electron withdrawing groups [31,57]. In total, the substituents were paired into 54 combinations, which could be categorized as donating-donating (D-D), withdrawing-withdrawing (W-W), and donating-withdrawing (D-W) pairs. Firstly, hydrogens on the R_1_ and R_1_’ sites were replaced. In subsequent calculations, dyes that exhibited the largest Δ**d** were doubled. The second pair of substituents were attached at the R_2_ and R_2_’ sites so that R_1_ = R_2_ and R_1_’ = R_2_’.

## 3. Results

### 3.1. Solvation Energies

**Figure 2:**
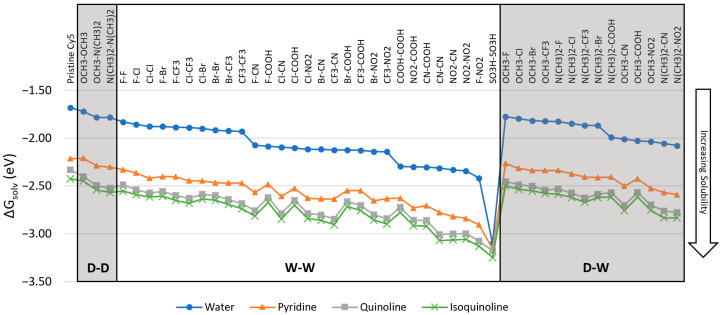
Solvation energies of substituted Cy5 dyes in water, pyridine, quinoline, and isoquinoline calculated using Equation (1). The geometry was optimized and the energies were calculated using the M06-2X functional. D-D is donating-donating, W-W is withdrawing-withdrawing, and D-W is donating-withdrawing. Substituted dyes are grouped according to substituent classification and ordered by decreasing ΔG_solv_. The lines added to the data are to highlight the trends of the data and are not meant to infer a quantitative behavior.

The calculated Gibbs free energies of solvation, ΔG_solv_, are shown in Figure 2 for implicit water, pyridine, quinoline, and isoquinoline solvents. Values of ΔG_solv_ were calculated to estimate the solubility of the dyes in the given solvent. The dyes are grouped according to their substituent’s classification and ordered from less negative to more negative ΔG_solv_ values in water. All ΔG_solv_ values were calculated using Equation (1) with the substituent pairs located at the R_1_ and R_1_’ positions of Cy5 (Figure 1). The calculations for ΔG_solv_, μ, and Δ**d** were conducted using the M06-2X and CAM-B3LYP functionals. It was found that both M06-2X and CAM-B3LYP yield similar values and the overall same trends for ΔG_solv_, μ, and Δ**d**, and so only the results for M06-2X are presented. For a comparison between M06-2X and CAM-B3LYP, see the Supporting Information.

Pristine Cy5 has the least negative ΔG_solv_. Like other studies [46,47], this indicates that pristine Cy5 is the most hydrophobic (i.e., least soluble). Many substituted Cy5 dyes containing D-D, W-W, and D-W pairs have comparable solubility to pristine Cy5, however, numerous substituent pairs make ΔG_solv_ more negative. A large number of W-W pairs exhibit the most negative solvation energies and are therefore taken to be the most hydrophilic (i.e., most aqueously soluble) and thus may hinder dye aggregation. However, numerous W-W pairs do not follow this trend, all of which are a combination of F, Cl, Br, and CF_3_ substituents. Specifically, the first 10 W-W pairs shown in Figure 2 exhibit ΔG_solv_ comparable to D-D and D-W pairs. The next 11 pairs have ΔG_solv_ similar to D-W pairs. The ΔG_solv_ values of the first 8 D-W pairs (which also contain F, Cl, Br, or CF_3_) are similar to those of the D-D pairs and the first 10 W-W pairs. The SO_3_H-SO_3_H-substituted Cy5 has the most negative ΔG_solv_ and is therefore the most soluble in water.

Comparing the values of ΔG_solv_ in water and other solvents, the dyes solvated in water exhibit less negative ΔG_solv_ than those in pyridine, quinoline, and isoquinoline. However, the substituents follow the same solubility trends as in water. In addition, the dyes prefer to form solutions in pyridine, quinoline, and isoquinoline due to the more negative ΔG_solv_ values of the dyes in these solvents compared to water. These three solvents are taken to mimic the structures of base pairs of DNA. It can therefore be inferred that substituted Cy5 dyes energetically prefer to be surrounded by DNA nucleobases (as mimicked by pyridine, quinoline, and isoquinoline) rather than to exist freely in aqueous solvent. Our predictions agree well with the molecular dynamics simulations conducted by Stennet et al. [58] and Cunningham et al. [22] in which cyanine dyes doubly linked to DNA duplexes solvated in water intercalated into the DNA structures to form dimers. Furthermore, the dyes with higher hydrophobicity may exhibit increased aggregation in DNA duplexes, leading to shorter dye-dye separations. This has been observed experimentally by Stadler et al. [28]. Our computational results suggest that D-D and D-W substituent pairs should exhibit denser dye packing than the other substituted dyes, resulting in comparatively enhanced dipole-dipole couplings, excitonic exchange energies, and two-body exciton interactions.

### 3.2. Dipole Moments

**Figure 3:**
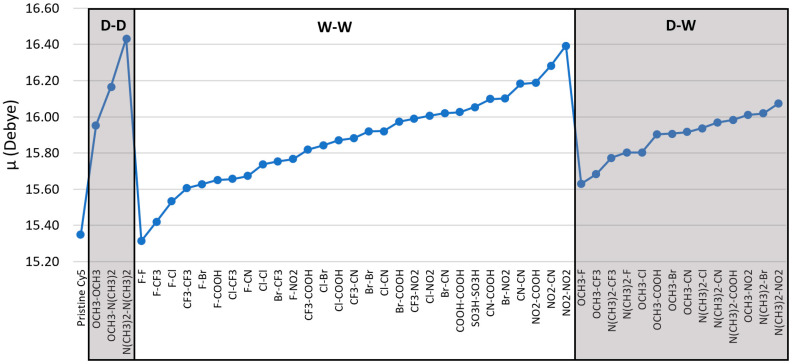
Transition dipole moment magnitudes (μ) of pristine and substituted Cy5 dyes. The given substituent groups are placed on R_1_ and R_1_’ sites in Figure 1. Geometry optimizations and excited state calculations were performed using the M06-2X functional. The dyes are grouped according to substituent classification and ordered by increasing μ. The lines added to the data are to highlight the trends of the data and are not meant to infer a quantitative behavior.

To determine the effects of electron donating and electron withdrawing substituents on the dipole properties of Cy5, DFT and TD-DFT methods were employed to calculate the μ and Δ**d** of pristine and substituted Cy5 dyes. The μ and Δ**d** shown were calculated using the M06-2X functional. The calculated value of μ for pristine Cy5 is 15.35 D, whose vector is primarily along the long axis (pentamethine chain) of the Cy5 dye. The calculated value of μ reasonably agrees with the experimental value of 13.4 D calculated from our colleague’s experimental monomer absorption data. Figure 3 shows that most of the substituent pairs increase μ, except the W-W pair F-F with a value of 15.32 D. N(CH_3_)_2_-N(CH_3_)_2_ has the largest μ of 16.43 D. Overall, the addition of substituents to the Cy5 dye has a minimal effect on μ—the largest change from pristine Cy5 is only 1.08 D. Since μ is relatively unaffected by the inclusion of different substituents, replacing H atoms with substituents in Cy5 should not decrease the excitonic exchange constant and may, in fact, increase it, which would be beneficial for excitonic applications.

**Figure 4:**
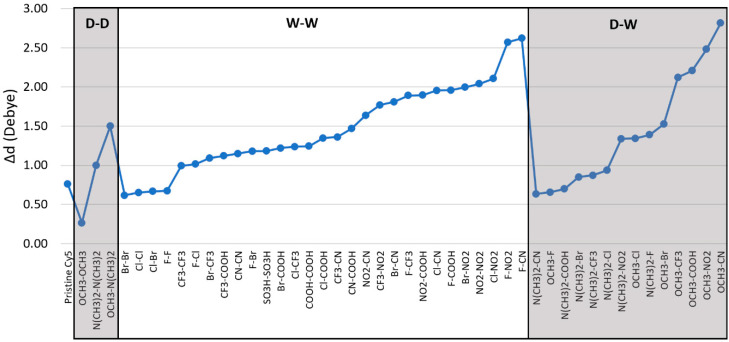
Static dipole difference magnitudes (Δ**d**) of pristine and substituted Cy5 dyes calculated using Equation (2). The given substituent groups are placed on R_1_ and R_1_’ sites in Figure 1. Ground state optimizations and excited state single point calculations were performed using the M06-2X functional. The dyes are grouped according to substituent classification and ordered according to increasing Δ**d**. The lines added to the data are to highlight trends and are not meant to infer a quantitative behavior.

Unlike μ, the addition of substituents on Cy5 has a greater effect on the static dipole difference magnitude, Δ**d**. Pristine Cy5 has a Δ**d** of 0.76 D. Figure 4 shows that multiple substituent pairs yield lower Δ**d** values than pristine Cy5, including the D-D pair OCH_3_-OCH_3_ with a Δ**d** of 0.27 D, the lowest Δ**d** of all substituent pairs tested. Four W-W pairs have lower Δ**d** than pristine Cy5, including Br-Br, Cl-Cl, Cl-Br, and F-F, with Δ**d** of 0.62–0.67 D. Three D-W pairs also yield lower Δ**d** than pristine Cy5, including N(CH_3_)_2_-CN, OCH_3_-F, and N(CH_3_)_2_-COOH with Δ**d** of 0.63–0.70. Furthermore, dyes with higher symmetry (i.e., dyes with two of the same substituents) consistently exhibit some of the lowest values of Δ**d**, an exception being the NO_2_-NO_2_ substituent pair.

Compared to pristine Cy5, most substituent pairs enhance Δ**d**. For W-W pairs, the largest Δ**d** belong to the pairs containing the strong electron withdrawing substituents NO_2_ and CN, where the F-CN pair exhibits the largest Δ**d** of the W-W pairs, 2.62 D. Many D-W pairs also yield larger Δ**d** than pristine Cy5, such as the pairs containing the OCH_3_ group, which is not the strongest electron donating substituent tested. Comparing W-W and D-W pairs, the substituent pair yielding the largest Δ**d** is OCH_3_-CN with a value of 2.82 D, more than triple the Δ**d** of pristine Cy5.

Overall, the addition of substituent pairs to pristine Cy5 can influence static dipole properties and increase static dipole differences. Recalling that an increase in Δ**d** leads to an increase in the two-body exciton interaction energy, the substitution of Cy5 with pairs of strong electron withdrawing substituents or pairs of strong electron donating substituents and withdrawing substituents can augment the two-body exciton interaction energy between dyes.

### 3.3. Double Substituents

Figure 3 and Figure 4 indicate that the substitution of Cy5 with pairs of substituents that have strong electron donating or strong electron withdrawing properties is consistent with an increase in both μ and Δ**d**. This finding led to the question as to would increasing the number of the same substituents further increase μ and Δ**d** and how would these substituents influence the dye’s solubility—i.e., ΔG_solv_. To investigate this question, the single substituents that were used on eight dyes that exhibited the largest Δ**d** values in Figure 4 were then doubled on those dyes. Specifically, the added substituents were placed on the R_2_ and R_2_’ sites on Cy5 (Figure 1).

**Figure 5:**
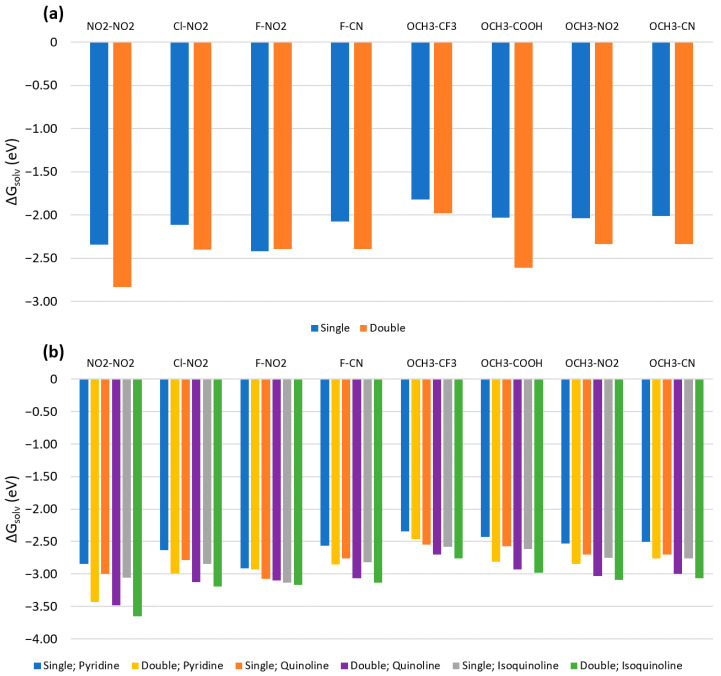
Gibbs free energy of solvation (ΔG_solv_) calculated using Equation (1) for singly and doubly substituted Cy5 dyes in (**a**) water and (**b**) pyridine, quinoline, and isoquinoline. Singly and doubly substituted Cy5 dyes were made by adding the given substituent pair to the R positions in Figure 1. For doubly substituted Cy5, two of the same substituent were added on the same side of the dye. All calculations were performed using the M06-2X functional.

Comparing the water solvation between single and double substituents shown in Figure 5a, ΔG_solv_ values for most of the double substituents are more negative, indicating that by doubling the number of substituents, the dyes become slightly more hydrophilic. However, the F-NO_2_ doubly substituted Cy5 has a less negative ΔG_solv_ by about 0.02 eV. The decreases in ΔG_solv_ are minimal, however, with changes ranging from 0.16 eV (OCH_3_-CF_3_) to 0.58 eV (OCH_3_-COOH).

Similar to the water solvated dyes, ΔG_solv_ values for doubly substituted Cy5 dyes in pyridine, quinoline, and isoquinoline all become slightly more negative, as shown in Figure 5b. This implies that while the dyes become more hydrophilic with double substitution, solvation in pyridine, quinoline, and isoquinoline is also slightly improved. Besides ΔG_solv_, other factors that were not considered, such as dye size or Coulombic effects between adjacent dyes or the dyes and DNA, may also influence dye intercalation into DNA.

**Figure 6:**
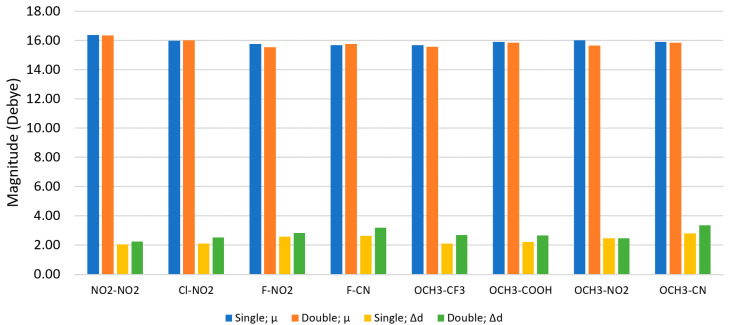
Magnitudes of transition dipole moments (μ) and static dipole differences (Δ**d**) for singly and doubly substituted Cy5 dyes. For doubly substituted Cy5, two of the same substituent were added on the same side of the dye. Ground state optimizations and excited state single point calculations to the first excited state were performed using the M06-2X functional.

For double substitutions, most of the calculated μ values slightly decrease, as shown in Figure 6. NO_2_-NO_2_ has the smallest decrease of 0.05 D, while OCH_3_-NO_2_ has the largest decrease of 0.36 D. Conversely, the double substitutions slightly increase Δ**d** in all cases except for OCH_3_-NO_2_, for which Δ**d** decreases by 0.02 D. The D-W pair OCH_3_-CF_3_ has the largest increase of 0.57 D. The D-W pair OCH_3_-CN also exhibits a large increase of Δ**d** (2.82 D to 3.35 D), over four times that of pristine Cy5. Of all dyes tested in this study, doubly substituted OCH_3_-CN has the largest Δ**d**.

### 3.4. Relationships with Hammett Constants

**Figure 7:**
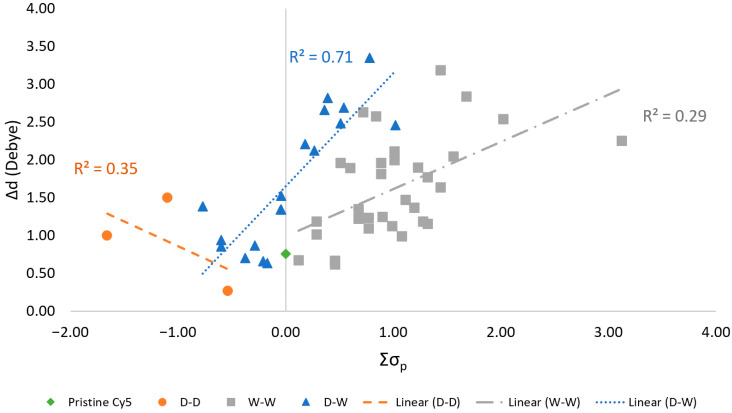
Static dipole difference magnitudes (Δ**d**) found using the M06-2X functional plotted against the sum of the Hammett constants of the substituents attached to Cy5 dyes. Linear fits of the separate sets of data are shown along with corresponding R^2^ values.

Recall that a substituent can be identified as either an electron donating or an electron withdrawing substituent based on the empirically derived σ_p_. Specifically, electron donating groups and electron withdrawing groups are associated with negative and positive values of σ_p_, respectively [31,57]. Hence, we hypothesize that there exists a relationship between the experimentally derived σ_p_ parameters and calculated Δ**d** values. For simplicity, we assume a linear correlation. To test this hypothesis, the Δ**d** of the dyes were plotted against the sum of the σ_p_ (Σσ_p_) of the substituents attached to the dyes, as shown in Figure 7. To determine the predictability of Δ**d** based on Σσ_p_, linear correlations are drawn for each data set (i.e., D-D, W-W, and D-W) and the variance between Δ**d** and Σσ_p_ is quantified with the coefficient of determination, R^2^. A perfect linear correlation corresponds to an R^2^ of 1 and no correlation corresponds to an R^2^ of 0. Plots of the Δ**d** values for D-D and W-W pairs against Σσ_p_ exhibit R^2^ values of 0.35 and 0.29, respectively. The D-W pairs exhibit a larger R^2^ value of 0.71. In general, within all three sets of data, the increase in the magnitude of Σσ_p_ values corresponds to a larger Δ**d**.

## 4. Discussion

Upon substitution of the hydrogen atoms at the ends of Cy5, μ remains relatively unaffected, with the largest change being 1.08 D, shown in Figure 3. However, the values for ΔG_solv_ and Δ**d** are altered compared to pristine Cy5, as shown in Figure 2 and Figure 4. Single atom W-W substituent pairs (F-F, Cl-Cl, Br-Br, F-Cl, F-Br, and Cl-Br) consistently exhibit less negative ΔG_solv_ (more hydrophobic) and lower Δ**d** than multi-atom W-W pairs. The symmetry of the substituted Cy5 dyes also contributes to the calculated values of Δ**d**, with more symmetric dyes (dyes with two of the same substituents) exhibiting smaller Δ**d** values than asymmetric dyes, overall. The W-W pair NO_2_-NO_2_ does not follow this trend, however, signifying that symmetry is not the only factor to consider for Δ**d**. Furthermore, asymmetry of substituted Cy5 does not always produce larger Δ**d** values compared to pristine Cy5, as is the case for numerous D-W pairs.

As shown in Figure 2, pristine Cy5 has the least negative ΔG_solv_, indicating that it is the least soluble in water, pyridine, quinoline, and isoquinoline. It is known that Cy5 and similar dyes exhibit limited solubility in water [59,60] and enhanced solubility in less polar solvents such as dimethyl sulfoxide (DMSO) and dimethyl formamide (DMF), both of which are used for experimental Cy5 sample preparation [61,62]. The relative solvation energies of Cy5 in water and the less polar pyridine, quinoline, and isoquinoline in the present study agree with the trend of Cy5 solubility in water, DMSO, and DMF. D-D and D-W substituted Cy5 dyes exhibit less negative ΔG_solv_ values compared to most W-W substituents. It is also found that the SO_3_H-SO_3_H substituted Cy5 has the most negative ΔG_solv_, which agrees with the experiments showing that SO_3_H substituents increase a molecule’s hydrophilicity [63]. The increase in hydrophilicity of the dyes can be attributed to the increase in polarity upon substitution. The polarity of the dyes in the ground state is estimated with their ground state dipole moments [46] (see Table S2). Upon substitution, the ground state dipole moments increase with respect to pristine Cy5 (except for N(CH_3_)_2_-N(CH_3_)_2_), indicating an increase in polarity and solubility. Compared to dyes solvated in water, dyes solvated in DNA base mimicking pyridine, quinoline, and isoquinoline solvents exhibit more negative ΔG_solv_, indicating that the dyes prefer to be solvated in those solvents rather than water. This may be correlated to the dye’s preference to intercalate into DNA structures, as observed in literature [7,22,28,58], rather than exist freely in an aqueous solution. However, this is a relatively simplistic view and further studies are ongoing.

Most substituent pairs exhibit larger values for Δ**d** than pristine Cy5, as shown in Figure 4. Similar trends for substituted symmetric molecules have been studied experimentally. When added to symmetric molecules, electron withdrawing substituents [26] and electron donating substituents [32,33] are suggested to induce large Δ**d** through charge separation and intramolecular charge transfer, shifting the charge density of the dyes. Two of the four largest Δ**d** values for W-W pairs belong to dyes with one of the weakest withdrawing substituents (F, Br, or Cl) paired with the strongest withdrawing substituent, NO_2_. The other largest Δ**d** values are for the F-CN substituted dye, with CN being the second strongest withdrawing substituent, and NO_2_-NO_2_. Interestingly, the four largest Δ**d** values for D-W substituted dyes do not belong to ones with the strongest donating substituent, N(CH_3_)_2_, but rather OCH_3_. The withdrawing substituents in the D-W pairs exhibiting the largest Δ**d**, however, are the strongest withdrawing substituents used. This indicates that withdrawing substituents have a larger effect on Δ**d**, as has been shown in a study on a similar system [64]. However, despite that the largest Δ**d** values for D-W pairs belong to dyes with OCH_3_, there is a positive linear correlation between Δ**d** and Σσ_p_ of D-W pairs with an R^2^ value of 0.71, as shown in Figure 7. Conversely, the three D-D pairs have a weak negative linear correlation with an R^2^ value of 0.35, where the Δ**d** values decrease as Σσ_p_ increases. W-W pairs also have a weak linear correlation, with an R^2^ value of 0.29. However, by visual inspection, as the magnitude of Σσ_p_ increases, so does Δ**d** for all pair types. For the case of W-W and D-W pairs, this implies that electron withdrawing substituents have a larger impact on Δ**d** for Cy5. The increase is greatest for D-W pairs and moderate for W-W pairs, as signified by the slopes of the linear fits.

Based on the results of this study, the addition of substituents can enhance excitonic properties of Cy5 aggregates. The inclusion of substituents decreases the value of ΔG_solv_ indicating that some substituents (most prevalently W-W substituents) make Cy5 more hydrophilic than others, thus hindering the aggregation of these dyes. Compared to most W-W pairs, D-D and D-W pairs are shown to be more hydrophobic, meaning aggregation may occur more readily for those dyes compared to W-W substituted Cy5. Furthermore, the inclusion of substituents does not degrade μ, indicating that the exciton exchange energy should remain relatively unaffected. W-W and D-W substituents can increase the Δ**d** of Cy5 by more than triple that of pristine Cy5. Assuming with a pristine Cy5 dimer orientation obtained from experiment [7,29] and that the exciton-exciton interaction energies increase with the square of Δ**d** [20], the exciton-exciton interaction energies of F-CN, F-NO_2_, OCH_3_-CN, and OCH_3_-NO_2_ substituted Cy5 dyes could be potentially increased by about ten times in comparison with that of the pristine Cy5 due to the increase in their Δ**d** values.

## 5. Conclusions

We performed DFT and TD-DFT calculations to determine the effects various substituents had on the ΔG_solv_, μ, and Δ**d** of Cy5. By substituting the hydrogens at the ends of a Cy5 dye, the ΔG_solv_ and Δ**d** of the dye can be altered. W-W substituent pairs were found to have the most negative values of ΔG_solv_ and therefore made Cy5 more soluble in water compared to D-D and D-W. Dyes solvated in pyridine, quinoline, and isoquinoline, taken to mimic DNA bases, were found to have more negative values than those solvated in water, suggesting that intercalation into DNA structures is favorable. Overall, the addition of substituents did not have a substantial impact on μ, with the largest difference between a substituted Cy5 and pristine Cy5 being 1.08 D. However, substituents did have a large impact on the Δ**d** of the dye. Numerous W-W and D-W substituent pairs increased Δ**d** by up to three times that of pristine Cy5. The W-W pair with the largest Δ**d** value was F-CN (2.62 D) and the D-W pair with the largest Δ**d** value was OCH_3_-CN (2.82 D). Doubling the substituents on the Cy5 dye did not appreciably make ΔG_solv_ more negative or increase Δ**d** overall, with the largest increase of Δ**d** being around 0.57 D for OCH_3_-CF_3_. The substituted Cy5 dye with the largest Δ**d** was doubly substituted OCH_3_-CN (3.35 D). Finally, correlations were made between the sum of experimentally derived σ_p_ parameters and Δ**d** values. Overall, an increase in the magnitude of Σσ_p_ of the substituents correlated to an increase in Δ**d**. This trend was more pronounced for D-W pairs than W-W pairs and D-D pairs. D-W pairs exhibited a positive linear correlation with an R^2^ of 0.71, signifying that the sum of σ_p_ values can be used to approximate the relative magnitudes of Δ**d** for substituted Cy5 dyes.

The substitution of Cy5 can enhance electronic properties for improved excitonic applications. D-D and D-W pairs have less negative ΔG_solv_ compared to W-W pairs, indicating that dye aggregation may be less favorable for W-W pairs. Furthermore, the substituents did not decrease μ of the dyes so that the excitonic exchange constant should not be degraded. Both W-W and D-W pairs increased Δ**d** and thus may enhance the two-exciton interaction term of the Frenkel Hamiltonian. The results of this study could help select the dye candidates with optimized electronic properties for desired applications. Specifically, these results will guide the synthesis and experimental studies for tailoring the dipole properties of Cy5 dyes via substituent engineering towards applications that exploit dye aggregates and exciton dynamics.

## Figures and Tables

**Table 1:** Substituents, their corresponding Hammett constants (σ_p_), and the classifications used in the current study. Negative values correspond to electron donating substituents and positive values correspond to electron withdrawing substituents.

Substituent	σ_p_	Classification
N(CH_3_)_2_	−0.83 ^a^	Donating
OCH_3_	−0.27 ^a^	
F	0.06 ^a^	Withdrawing
Cl	0.23 ^a^	
Br	0.23 ^a^	
COOH	0.45 ^a^	
CF_3_	0.54 ^a^	
SO_3_H	0.64 ^b^	
CN	0.66 ^a^	
NO_2_	0.78 ^a^	

^a^ Values taken from Hansch et al. [55]. ^b^ Values taken from Imaizumi et al. [56].

## Data Availability

Select solvation energy and dipole moment data presented in this study are available in the Supporting Information. In the near-term, all other data presented are available upon request from the corresponding author. Within six months, we plan to make all data available in a publicly accessible data repository.

## Supplementary Materials

The following are available online. Figure S1: Gibbs free energies of solvation (ΔG_solv_) for pristine and substituted Cy5 using the M06-2X functional calculated using the Gibbs free energies of the dyes in solvent and vacuum (instead of total energies), Figure S2: Gibbs free energies of solvation (ΔG_solv_) for pristine and substituted Cy5 using the M06-2X and CAM-B3LYP functionals, Figure S3: Transition dipole moments (μ) for pristine and substituted Cy5 using the M06-2X and CAM-B3LYP functionals, Figure S4: Static dipole moment differences (Δ**d**) for pristine and substituted Cy5 using the M06-2X and CAM-B3LYP functionals, Table S1: Solvation energies of pristine and substituted Cy5 dyes in water, pyridine, quinoline, and isoquinoline solvents, Table S2: Magnitudes of the ground state dipole moments (GSDM) and excited state dipole moments (ESDM) calculated for each Cy5 dye with the M06-2X and CAM-B3LYP functionals.

## Sample Availability

Samples of the compounds are not available from the authors.
