# Supplementary material for: Substituent Effects on the Solubility and Electronic Properties of the Cyanine Dye Cy5: Density Functional and Time-Dependent Density Functional Theory Calculations
Source: Molecules. 2021 Jan 20;26(3):524. doi: 10.3390/molecules26030524 (PMC7863957; doi:10.3390/molecules26030524)
Supplement: Supplementary file 1 [file molecules-26-00524-s001.pdf]

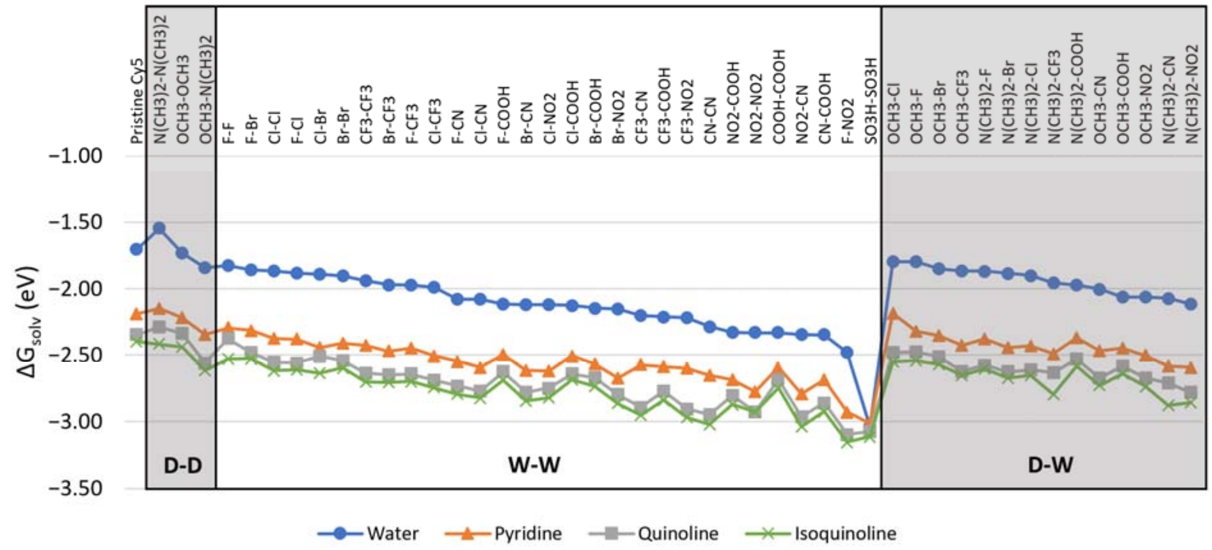

Figure S1. Gibbs free energies of solvation (ΔG_{solv}) for pristine and substituted Cy5 calculated with the M06-2X functional using Equation 1. The Gibbs free energies of the dyes in solvent and vacuum were calculated at 298K, as opposed to the total energies displayed in the main text. Values in each section are ordered according to increasing ΔG_{solv} in water. Lines added to the data are to highlight trends and not meant to infer quantitative behavior. D-D is donating-donating, W-W is withdrawing-withdrawing, and D-W is donating-withdrawing.

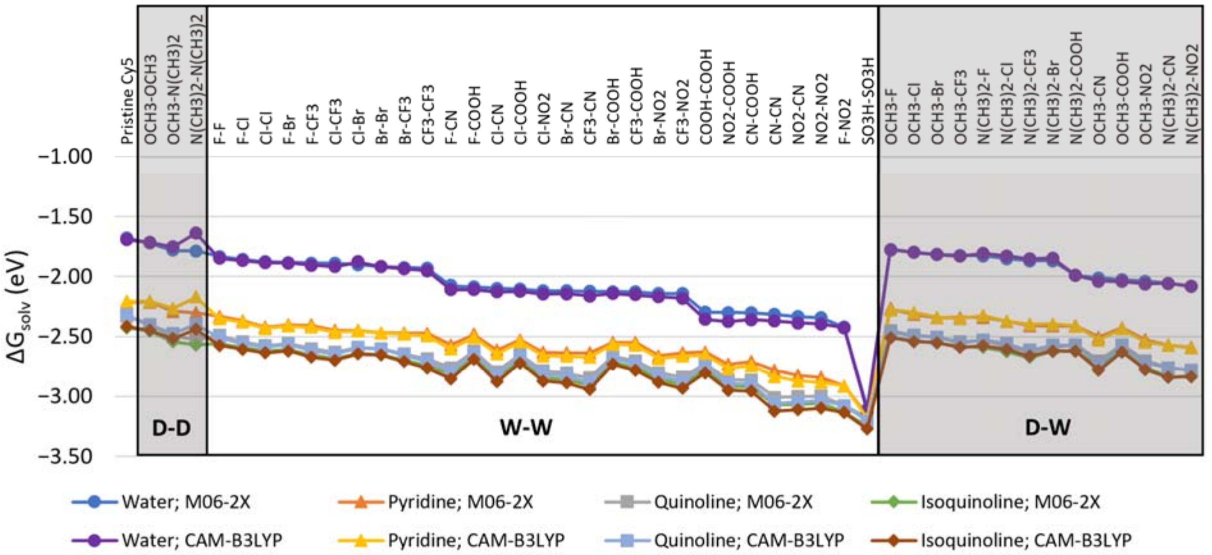

Figure S2. Gibbs free energies of solvation (ΔG_{solv}) for pristine and substituted Cy5 calculated using the M06-2X and CAM-B3LYP functionals and Equation 1. The total energies (as opposed to the Gibbs free energies) were used. Values in each section are ordered according to increasing ΔG_{solv} calculated with M06-2X. Lines added to the data are to highlight trends and not meant to infer quantitative behavior. D-D is donating-donating, W-W is withdrawing-withdrawing, and D-W is donating-withdrawing.

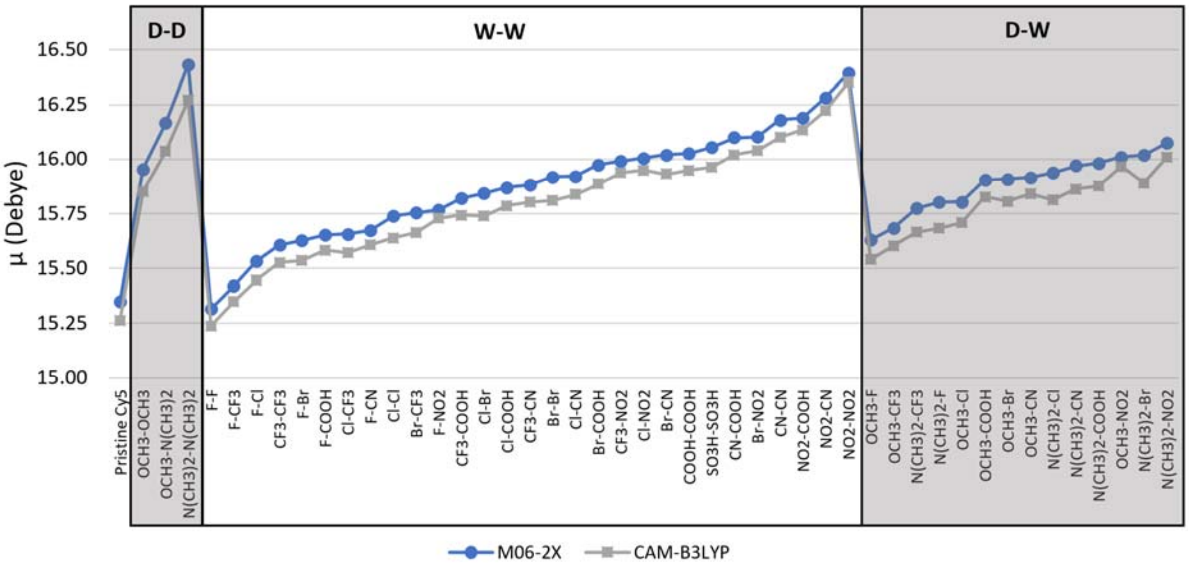

Figure S3. Transition dipole moments (μ) for pristine and substituted Cy5 calculated using the M06-2X and CAM-B3LYP functionals. Values in each section are ordered according to increasing μ calculated with M06-2X. Lines added to the data are to highlight trends and not meant to infer quantitative behavior. D-D is donating-donating, W-W is withdrawing-withdrawing, and D-W is donating-withdrawing.

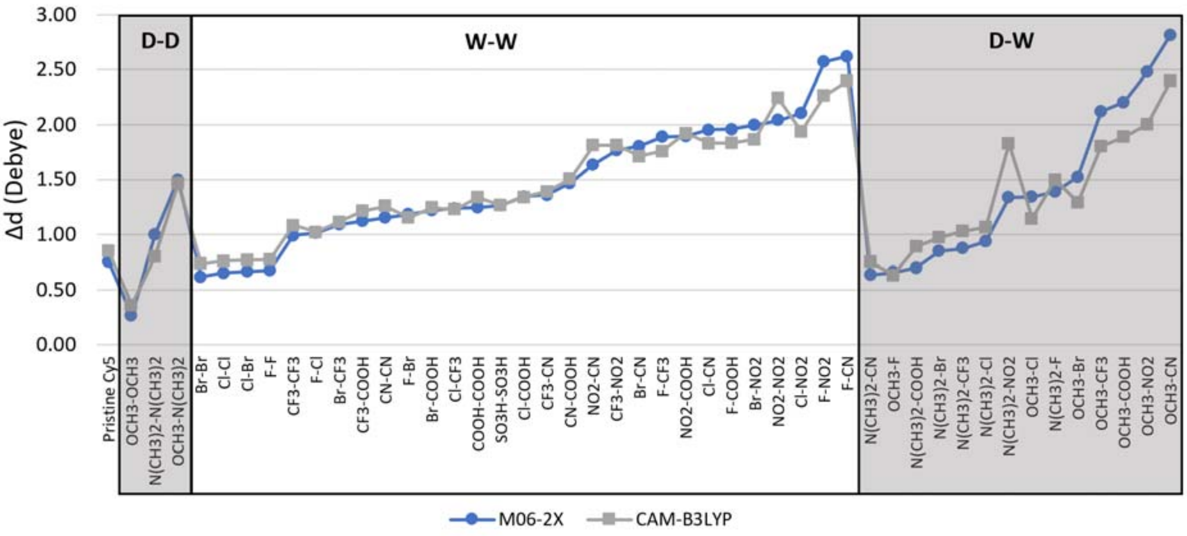

Figure S4. Static dipole moment differences (Δd) for pristine and substituted Cy5 using the M06-2X and CAM-B3LYP functionals calculated using Equation 2. Values in each section are ordered according to increasing Δd calculated with M06-2X. Lines added to the data are to highlight trends and not meant to infer quantitative behavior. D-D is donating-donating, W-W is withdrawing-withdrawing, and D-W is donating-withdrawing.

Table S1. Solvation energies of pristine and substituted Cy5 dyes in water, pyridine, quinoline, and isoquinoline solvents. Geometry relaxations and energies were determined using the M06-2X functional.

System	Water (eV)	Pyridine (eV)	Quinoline (eV)	Isoquinoline (eV)
**Pristine Cy5**	-1.68	-2.22	-2.33	-2.43
**Single Substitutions**				
**OCH3-OCH3**	-1.72	-2.21	-2.40	-2.45
**N(CH3)2-N(CH3)2**	-1.78	-2.30	-2.53	-2.57
**OCH3-N(CH3)2**	-1.78	-2.29	-2.50	-2.54
**F-F**	-1.83	-2.33	-2.49	-2.56
**F-Cl**	-1.86	-2.37	-2.54	-2.59
**F-Br**	-1.88	-2.40	-2.56	-2.61
**F-CF3**	-1.89	-2.41	-2.60	-2.65
**F-NO2**	-2.42	-2.91	-3.08	-3.13
**F-CN**	-2.08	-2.57	-2.76	-2.82
**F-COOH**	-2.09	-2.48	-2.62	-2.67
**Cl-Cl**	-1.88	-2.42	-2.57	-2.62
**Cl-Br**	-1.90	-2.45	-2.59	-2.64
**Cl-CF3**	-1.89	-2.45	-2.63	-2.68
**Cl-NO2**	-2.12	-2.63	-2.79	-2.84
**Cl-CN**	-2.10	-2.61	-2.79	-2.85
**Cl-COOH**	-2.11	-2.53	-2.65	-2.70
**Br-Br**	-1.92	-2.47	-2.60	-2.65
**Br-CF3**	-1.92	-2.47	-2.64	-2.69
**Br-NO2**	-2.14	-2.66	-2.80	-2.86
**Br-CN**	-2.12	-2.64	-2.80	-2.86
**Br-COOH**	-2.13	-2.55	-2.66	-2.72
**CF3-CF3**	-1.93	-2.47	-2.69	-2.74
**CF3-NO2**	-2.14	-2.64	-2.84	-2.90
**CF3-CN**	-2.12	-2.64	-2.85	-2.91
**CF3-COOH**	-2.13	-2.55	-2.71	-2.76
**NO2-NO2**	-2.34	-2.84	-3.00	-3.06
**NO2-CN**	-2.33	-2.82	-3.00	-3.06
**NO2-COOH**	-2.30	-2.73	-2.86	-2.92
**CN-CN**	-2.32	-2.78	-3.01	-3.07
**CN-COOH**	-2.30	-2.71	-2.86	-2.92
**COOH-COOH**	-2.30	-2.63	-2.73	-2.78
**OCH3-F**	-1.77	-2.27	-2.45	-2.50
**N(CH3)2-F**	-1.83	-2.34	-2.53	-2.59
**OCH3-Cl**	-1.80	-2.32	-2.49	-2.54
**N(CH3)2-Cl**	-1.85	-2.37	-2.58	-2.62

**OCH3-Br**	-1.82	-2.34	-2.50	-2.55
**N(CH3)2-Br**	-1.87	-2.41	-2.59	-2.62
**OCH3-CF3**	-1.82	-2.34	-2.54	-2.58
**N(CH3)2-CF3**	-1.87	-2.41	-2.63	-2.68
**OCH3-NO2**	-2.04	-2.53	-2.70	-2.75
**N(CH3)2-NO2**	-2.08	-2.59	-2.78	-2.83
**OCH3-CN**	-2.01	-2.50	-2.70	-2.76
**N(CH3)2-CN**	-2.06	-2.57	-2.76	-2.84
**OCH3-COOH**	-2.03	-2.42	-2.57	-2.62
**N(CH3)2-COOH**	-1.99	-2.41	-2.57	-2.62
**SO3H-SO3H**	-3.10	-3.16	-3.18	-3.25
**Double Substitutions**				
**NO2-NO2**	-2.83	-3.43	-3.48	-3.65
**Cl-NO2**	-2.40	-2.99	-3.13	-3.19
**F-CN**	-2.39	-2.85	-3.07	-3.13
**F-NO2**	-2.40	-2.93	-3.10	-3.16
**OCH3-CF3**	-1.98	-2.46	-2.70	-2.76
**OCH3-COOH**	-2.61	-2.81	-2.93	-2.98
**OCH3-NO2**	-2.34	-2.85	-3.03	-3.09
**OCH3-CN**	-2.33	-2.76	-3.00	-3.06

Table S2. Magnitudes of the ground state dipole moments (GSDMs) and excited state dipole moments (ESDMs) calculated for pristine and substituted Cy5 dyes with the M06-2X and CAM-B3LYP functionals.

**System**	**M06-2X**		**CAM-B3LYP**	
	**GSDM (Debye)**	**ESDM (Debye)**	**GSDM (Debye)**	**ESDM (Debye)**
**Pristine Cy5**	3.65	4.41	3.61	4.46
**Single Substitutions**				
**OCH3-OCH3**	6.66	6.92	6.65	7.01
**N(CH3)2-N(CH3)2**	2.21	1.21	2.21	1.41
**OCH3-N(CH3)2**	4.60	4.74	4.56	4.79
**F-F**	7.81	8.48	7.83	8.60
**F-Cl**	8.99	9.44	8.97	9.54
**F-Br**	11.28	11.37	11.16	11.40
**F-CF3**	13.58	13.32	13.46	13.40
**F-NO2**	16.91	16.70	16.83	17.11
**F-CN**	13.79	13.36	13.86	13.66
**F-COOH**	11.18	11.50	11.20	11.70
**Cl-Cl**	9.34	9.99	9.32	10.08
**Cl-Br**	10.76	11.32	10.67	11.35
**Cl-CF3**	12.73	13.11	12.64	13.17

**Cl-NO2**	15.81	16.24	15.76	16.59
**Cl-CN**	13.14	13.42	13.24	13.68
**Cl-COOH**	11.07	11.90	11.13	12.07
**Br-Br**	11.13	11.75	11.00	11.73
**Br-CF3**	12.51	13.19	12.42	13.21
**Br-NO2**	15.07	15.95	15.03	16.21
**Br-CN**	13.21	13.95	13.26	14.11
**Br-COOH**	11.88	12.92	11.90	13.02
**CF3-CF3**	13.49	14.48	13.43	14.52
**CF3-NO2**	15.48	16.90	15.48	17.11
**CF3-CN**	14.26	15.38	14.33	15.54
**CF3-COOH**	13.52	14.60	13.56	14.74
**NO2-NO2**	16.63	18.67	16.67	18.92
**NO2-CN**	16.24	17.81	16.33	18.13
**NO2-COOH**	16.21	17.40	16.29	17.76
**CN-CN**	14.85	16.00	15.07	16.32
**CN-COOH**	14.06	15.06	14.26	15.37
**COOH-COOH**	12.43	13.67	12.59	13.93
**OCH3-F**	8.00	8.23	8.02	8.40
**N(CH3)2-F**	6.90	7.54	6.82	7.67
**OCH3-Cl**	10.12	9.77	10.09	9.96
**N(CH3)2-Cl**	9.49	9.78	9.32	9.90
**OCH3-Br**	13.04	12.32	12.90	12.46
**N(CH3)2-Br**	12.73	12.97	12.45	13.00
**OCH3-CF3**	15.51	14.44	15.38	14.63
**N(CH3)2-CF3**	15.26	15.52	14.98	15.57
**OCH3-NO2**	19.05	18.22	18.97	18.79
**N(CH3)2-NO2**	18.94	20.06	18.71	20.37
**OCH3-CN**	15.57	14.22	15.64	14.65
**N(CH3)2-CN**	15.20	15.09	15.12	15.41
**OCH3-COOH**	12.56	11.92	12.55	12.20
**N(CH3)2-COOH**	11.98	12.14	11.82	12.31
**SO3H-SO3H**	21.11	22.25	21.12	22.34
**Double Substitutions**				
**NO2-NO2**	13.21	15.46	13.18	15.65
**Cl-NO2**	18.02	17.67	17.80	18.12
**F-CN**	16.51	14.98	16.55	15.36
**F-NO2**	21.02	20.03	20.71	20.52
**OCH3-CF3**	20.40	18.61	20.19	18.82
**OCH3-COOH**	17.67	16.35	17.62	16.68
**OCH3-NO2**	24.65	23.68	24.38	24.39
**OCH3-CN**	20.13	18.05	20.21	18.63
